# Government Inaction on Ratings and Government Subsidies to the US Film Industry Help Promote Youth Smoking

**DOI:** 10.1371/journal.pmed.1001077

**Published:** 2011-08-23

**Authors:** Christopher Millett, Jonathan R. Polansky, Stanton A. Glantz

**Affiliations:** 1 Department of Primary Care and Public Health, Imperial College London, London, United Kingdom; 2 South Asia Network for Chronic Disease, Public Health Foundation of India, New Delhi, India; 3 Onbeyond LLC, Fairfax, California, United States of America; 4 Division of Cardiology, University of California, San Francisco, California, United States of America; 5 Center for Tobacco Control Research and Education, University of California, San Francisco, California, United States of America

## Abstract

Christopher Millett and colleagues examine government inaction on the WHO recommendation for adult content ratings in films with smoking, and highlight the generous film industry subsidies these countries provide.

**Summary Points**

- Exposure to tobacco imagery in movies is a potent cause of youth experimentation and progression to established smoking.
- The World Health Organization and US Centers for Disease Control and Prevention, among others, recommend that all future movies with scenes of smoking (and other tobacco) be given an adult content rating.
- This recommendation has not yet been widely implemented. Even more problematic, many governments provide generous subsidies to the US film industry to produce youth-rated films that contain smoking and as such indirectly promote youth smoking.
- Between one-half and two-thirds of US-produced films that are youth-rated and government-subsidized in Britain, Canada, or the US contain smoking.
- Government subsidies for top-grossing films with smoking rival or surpass public spending on tobacco prevention campaigns in Britain and more than a dozen US states.
- Governments should ensure that film subsidy programmes are harmonised with public health goals by making films with tobacco imagery ineligible for public subsidies.

## Introduction

Research indicates that exposure to tobacco imagery in movies is a potent cause [1] of youth experimentation and progression to established smoking [2]–[4], with a dose-response relationship that indicates heavily exposed youths are about three times as likely to begin smoking as lightly exposed youths [1]. Links between exposure to tobacco imagery in movies and initiation of smoking among youth have been documented in several countries with distinct cultures, diverse tobacco regulatory regimes (including varying controls on advertising), and different smoking prevalences [5]–[8]. This evidence led the World Health Organization (WHO) to recommend [2] as part of implementing Article 13 of the WHO Framework Convention on Tobacco Control (FCTC) [9] that all future movies with scenes of smoking (and other tobacco) be given an adult content rating, with the possible exception of movies that depict the dangers of tobacco use or smoking by an actual historical figure who actually smoked.

The primary logic for recommending an adult content rating policy is to create an economic incentive for producers to leave smoking out of movies that are marketed to youths. A 2005 study in the US concluded that the return on investment for youth-rated movies was 70%, compared with 29% for adult content (R-rated) movies [10]. Essentially eliminating smoking and other tobacco imagery from youth-rated films would substantially reduce the total exposure of onscreen smoking images delivered to youth. (In addition, while youth do see some adult-rated films, they are less likely to see them than youth-rated films.)

This adult rating recommendation has not yet been widely adopted. Even more problematic, many governments provide generous subsidies to the US film industry to produce youth-rated films that contain smoking and as such indirectly promote youth smoking.

This paper describes, firstly, the status of implementing the WHO recommendation on adult content ratings in Great Britain, Canada, and the US. Secondly, it examines how film industry subsidies are administered in these countries, including the magnitude of subsidies for youth-rated films containing smoking, and compares these subsidies with spending on tobacco control programmes.

## Exposure Levels and Implications for Youth Smoking

Because of different film rating practices among the British Board of Film Classification (BBFC), Canadian provincial film boards, and the Motion Picture Association of America (MPAA), more films with more tobacco incidents (defined as the appearance of tobacco use, a tobacco product, or a tobacco brand trademark) are in films rated for adolescents in the UK and Canada than the US. Between 2001 and 2006, 79% (150/190) of films rated for adults in the US (R, under 17 admitted only with parent or guardian) were rated as suitable for youths in the UK (PG, parental guidance; 12/12A, under 12 admitted only with an adult; 15, accessible to youth 15 and older), as were 60% (30/50) in Canada (PG, parental guidance; 14A, under 14 admitted only with adult) during 2009 [11],[12]. As a consequence, 87% (3,308/3,808) and 75% (1,444/1,935) of onscreen tobacco incidents in top-grossing (predominately US) films released in the UK and Canada were youth rated compared to 46% (7,538/16,325; 2002–2006) and 44% (856/1,935; 2009) in the US [11],[12]. Because of these differences in rating practices Canadian and British youth are exposed to higher levels of tobacco imagery in films that are rated for and marketed directly to youth than their US counterparts, with the bulk of exposure coming from films financed and distributed by US media conglomerates [11]–[13].

The actual population-level impact of smoking in a given film will depend on how successful it is. This impact is quantified by computing the number of tobacco “impressions” each film delivers (one impression is one person seeing one tobacco incident one time). The percentage of youth-rated tobacco impressions attributable to top-grossing films from the major US studios was 93% (4.2 billion/4.5 billion) in the UK (2001–2006) [11], 76% (872 million/1.2 billion) in Canada (2009) [12], and 87% (46.5 billion/53.5 billion) in the US between 2005–2009 [14].

In 2006, an estimated 417,000 British youths aged 11–15 years were ever smokers and 194,000 youth were regular smokers [15], as were 134,000 Canadian (15–19 years), and 1.1 million US youth (12–17 years) due to exposure to tobacco imagery in movies [12],[16]. The estimated numbers of British and Canadian youth who started smoking because of exposure to onscreen tobacco use are likely to be low because they are based on a population attributable risk fraction (0.44; 95% confidence interval [CI] 0.34–0.58) (after adjusting for other factors known to influence tobacco initiation in youth including parental smoking behaviour, peer influence, and rebelliousness) derived from US studies [15] and because UK and Canadian youth are subjected to higher levels of exposure to onscreen smoking than US youth. In addition, because the UK and Canada have much more stringent restrictions on direct cigarette advertising and promotion than the US, the relative contribution of onscreen smoking exposure to smoking initiation and progression to established smoking would be expected to be higher in Britain and Canada than the US [15].

## Government Inaction on Adult Ratings for Films with Smoking

### Britain

In Britain, the Labour government published a tobacco control strategy for England in February 2010, a key objective of which was to “stop the inflow of young people recruited as smokers” [17]. As part of this strategy the government recommended that smoking “must not be featured in programmes made primarily for children (defined as <15 years of age) unless there is strong editorial justification” and smoking “must not be condoned, encouraged or glamorised in other programmes likely to be widely seen or heard by under-18 s unless there is editorial justification.” These recommendations fell well short of actions proposed by the WHO. In particular, by only calling for restrictions on films that “feature” smoking that is “encouraged or glamorized” unless there is “strong editorial justification,” the government allows for smoking in virtually any film because such terms are so subjective as to be undefined. In 2010, the BBFC mentioned smoking in its online “Extended Classification Information” for 23.6% (13/59) of all top-grossing US films released in Britain with smoking, while rating 93% (55/59) of these films as suitable for youth. Only one film's publicly displayed “Consumer Advisory” noted its smoking content. The British coalition government that took power in 2010 published a new tobacco control strategy in March 2011 in which they commit to “continue to work to reduce the depiction of smoking in the media, including through bringing together media regulators and the entertainment industry to consider what more can be done” [18]. Like their predecessor Labour government, such a step is unlikely to have any meaningful effect on the levels of youth exposure to smoking in youth-rated films.

### Canada

As of May 2011 smoking was not yet part of the film classification criteria in any Canadian province, but the public health community was pressing the issue with several provincial rating authorities and this proposal is under consideration by at least one government agency. In 2010 the Ontario Ministry of Health Promotion and Sport's Smoke-Free Ontario Scientific Advisory Committee recommended that films and video games with tobacco imagery receive an adult rating (18A) [19]. Ontario's provincial Film Review Board (OFRB) reports films' smoking status in online-only “Detailed Observations,” not publicly in ratings descriptors. The OFRB listed “tobacco use” for 77% (46/60) of top-grossing US films released in Canada during 2010 with known smoking content and rated 82% (49/60) of these as appropriate for youth: PG, 12A, or 14A.

### US

In the US, leading public health and medical organizations have repeatedly called for R-rating future films with smoking. The US Centers for Disease Control and Prevention (CDC) has also recommended the adult rating as an effective method for reducing youth exposure to onscreen smoking, because it would create a market incentive to keep films designed to be marketed to youth (by obtaining a youth rating) tobacco free [20],[21]. Persistent public pressure has caused the US film industry to reduce smoking incidents since 2005 in both youth- and adult-rated films [19], although progress has been inconsistent across media companies, with Time Warner, Disney, and Comcast (Universal) nearly eliminating smoking from their youth-rated films in 2010 and News Corp (Fox), Viacom (Paramount), Sony, and the independent studios showing much smaller reductions [21]. However, the US MPAA, the lobbying organization for the major US studios that governs the US' voluntary rating system, refused to make smoking a rating criterion in 2007, instead adding a fine-print label to a small fraction of the youth ratings for films with smoking released each year [22]. For the sample of 2010 films with smoking also released in the UK and Canada, the MPAA listed “smoking” in just 10% (6/60) of the small-print “descriptors” publicly associated with the film's rating. In the US, 45% (27/60) of top-grossing 2010 films with smoking were rated for youth: PG or PG-13. None of the 13 films with 50 or more tobacco incidents, including the six that were youth rated, carried an MPAA “smoking” descriptor. Since 2007, the MPAA has never identified a film that was rated R because of its smoking content.

Compounding the lack of government inaction on the WHO FCTC and CDC recommendations to provide adult film ratings for films with smoking is the extent to which these same governments subsidize the US film industry, which serves to indirectly promote youth smoking.

## Government Subsidies to Youth Rated Films with Smoking

### Britain

Government intervention to attract US studio productions to sustain the British film industry started in the 1970s and continued through Conservative and Labour governments [23]. Under 2007 rules, a certified “British” film (meeting the EU-approved “Cultural Test” [24]) that spends at least one-quarter of a minimum production budget of £20 million (US$31 million) in Britain receives an effective tax relief of 16% against its British spend; “limited-budget” films below £20 million receive 20% relief [25]. Film productions developed by US companies received an estimated three-quarters of the value of available UK tax credits between 2006 and 2008 [26]. Tax credits are not the only subsidy for film production in the Britain. Additional subsidies estimated at £50 million (US$78 million) annually—including National Lottery funds channelled through the UK Film Council, local economic development funds, and European Union grants—are invested in “British” films [26]. Although the Coalition government elected in 2010 abolished the UK Film Council, it has publicly stated that, despite proposed cuts to some public health programmes [27], subsidies to the US film industry would continue [28].

Data from the UK Film Council indicate that 15% (144/988) of “British” films made between 2003 and 2009 were produced by US film companies [26]. Of the 144 US films certified “British,” the tobacco content of 102 films is known because they ranked among the top ten grossing films in any week after their theatrical release and so their tobacco content has been monitored [27]. Of these 102 films, 67 feature tobacco imagery (Table 1).

**Table 1:** US-produced “British” films with tobacco imagery, by UK film classification, 2003–2009.

BBFC Rating	Number of Films^a^	With Tobacco Imagery	Percent with Imagery
U/PG	28	17	61
12A	35	20	57
15	31	23	74
18	5	5	100
**Total**	99	65	66

a The BBFC did not classify three “British” film titles rated by the Motion Picture Association of America—*The Eagle of the Ninth* (smoke free), *Scoop* (smoking), and *Doom* (smoking)—and they are omitted from this table.

We used the individual films' production budget estimates published by authoritative film industry database (http://www.IMDbPro.com/) and UK Film Council data on the fraction of total budget typically spent in Britain by “inward investment,” “domestic,” and “coproduction” films to calculate each US “British” film's tax credit (Table 2). These data suggest that, between 2003 and 2009, £338 million (US$524 million) of Film Tax Credits awarded for film production in Britain went to US-produced “British” films with tobacco imagery, almost all of which are rated for children and adolescents. The annual direct cost to the Exchequer of £48 million (US$74 million) over this 7-year period is double that spent—£23 million (US$36 million)—by the UK government in 2008–2009 on mass media health promotion campaigns to avert young people from starting to smoke and support adults to quit (Table 2) [29].

**Table 2:** Estimated UK Film Tax Credits awarded to US-produced films, by BBFC rating and tobacco content, 2003–2009.

Content	Rating	Number of Films^a^	Tax Credit (£ millions)	Percent
Smoking	Youth rated	55	318	43
	Adult rated	4	20	3
Smoke free	Youth rated	30	407	55
	Adult rated	0	0	0
**Total**		89	745	100

a Of the 99 US-produced “British” films with known tobacco content that were rated by BBFC, ten lacked published production budget estimates on which film credit calculations are based and have been omitted from this table.

### Canada

Canada expanded subsidies to the US film industry in 1998 through provincial level Production Services Tax Credits and generous federal tax relief. These subsidies have helped attract US-produced films: Canada was the location for half of US-produced films shot outside the US between 2004 and 2009 [12]. Of films shot in Canada over this period that reached top-grossing status in the domestic (Canada-US) film market, 89% (131/148) were produced and distributed by major US studios. Analysis of programme data from British Columbia and Ontario, together accounting for 75% of US film production in Canada, indicate that these provinces awarded CDN$41 million (US$40 million) in Production Services Tax Credits to non-Canadian (i.e., US) feature film projects in fiscal year 2008–2009 [12]. These projects also qualified for a 16% federal labour tax credit, bringing the total public subsidy for films shot in these two provinces to CDN$60 million (US$59 million)—equating to CDN$80 million (US$79 million) nationally.

Between 2004 and 2009, 80% (119/148) of US-produced films shot in Canada were youth rated by provincial governments and 52% (62/119) of subsidised youth-rated films contained smoking (Table 3). (While every province applies its own rating, there was 80% (115/148) unanimity among the provinces in 2009 ratings, overall, and 89% (62/70) agreement on whether films should be adult rated or youth accessible [12]). These data suggest that approximately CDN$32 million (US$31 million) of annual public funding were used during fiscal year 2008–2009 to subsidise youth-rated US studio films, shot in Canada, that contain smoking. Canada spent a total of CDN$150 million (US$147 million) on tobacco control during fiscal year 2009–2010 [30].

**Table 3:** US-produced Canadian located films with tobacco imagery, by Canadian film classification, 2004–2009.

Rating	Number of Films	With Tobacco Imagery	Percent with Imagery
G/PG	78	33	42
14A	41	29	71
18A	29	20	69
**Total**	148	82	55

### US

Forty US states collectively offered US$1.3 billion in film and video “production incentives” to the US film industry in 2008 [16]. These grants, commonly in the form of tax credits, cover 25% of Hollywood's day-to-day production costs in the US on average [16]. Of the total subsidies offered, some are not taken up by the film industry because states lack production infrastructure. TV series and undistributed low-budget films also draw from the subsidy pool. For top-grossing films, produced in the US and released nationally and internationally, 16 states provided an annual average of US$436 million 2008–2010.

Two thirds (67%) of these annual subsidies were provided by just five states: New York (US$100 million), California (US$70 million), Louisiana (US$40 million), Massachusetts (US$40 million), and Pennsylvania (US$40 million). In 2010, US$288 million (66%) of subsidies to top-grossing films went to films with smoking, including US$127 million (30%) to youth-rated films. The 16 states subsidizing top-grossing films with smoking spent more on that activity in 2010 (US$288 million) than they budgeted for 2011 tobacco control programs (US$280 million) [31]. A case study of the state of Louisiana is contained in Box 1.

**Box 1. Case Study: Louisiana**

In 2010, Louisiana offered a 30% tax credit against all in-state production expenditures and an additional 5% payroll tax credit for employment of state residents. Out-of-state film producers with no Louisiana income tax liability can sell their tax credits back to the state or to a large state taxpayer, converting the tax credit into a cash grant worth more than 25% of a film's production costs. Film projects spending the minimum required for eligibility (US$300,000) do not need to be completed or released to the public in order to earn the tax credit [38].

To determine how many recent Louisiana feature film productions eligible for taxpayer subsidies featured tobacco imagery and how much subsidy they may have received, we used project listings provided by Louisiana Economic Development (LED) and IMDbPro.com to identify feature films from 2006 through 2008 released nationally to commercial theatres. (Louisiana is one of the few states that will release detailed information on its film subsidies.) From 2006 through 2008, Louisiana certified 93 feature films as eligible for production tax credits totalling US$211 million. Of these 93 films, 27 (29%) had been released to theatres nationally by the end of 2010, with tax credits estimated at US$129 million. Another 20 films (22%) went straight to DVD with US$31 million in tax credits. The remaining 46 films (49%), certified for US$51 million in state tax credits, were not commercially distributed in any form. Of the 27 wide-release films whose smoking content is known, 17 (63%) featured tobacco; of the 17 wide-release films that were youth rated, eight (47%) contained smoking (Table 4). From 2006 to 2008, Louisiana spent more than three times as much subsidizing commercial films including smoking than it spent on tobacco prevention and control programs (US$85 million versus US$26.3 million) [39]–[41].

## Implications for Policy

The failure of governments to implement the WHO recommendation that an adult rating (18 in UK, 18A in Canada, and R in US) be assigned to films that contain tobacco imagery [2] means that rating authorities in all three countries still certify large numbers of films containing smoking and other tobacco imagery as appropriate for youth despite strong scientific evidence that exposure to these tobacco images causes youth smoking [1],[3],[4]. In addition, adults are also exposed to smoking in youth-rated films and their smoking behaviour is also affected [32]–[34]. Thus, a positive side effect of protecting youth from images of smoking in youth-rated films will also have benefits for adults.

Beyond continuing to allow rating systems that certify large numbers of films with tobacco use as appropriate for children, governments in UK and most Canadian provinces and US states go further by indirectly promoting smoking to youth through the provision of generous subsidies to the US movie industry [11],[21],[35],[36]. Our analysis suggests that between one-half and two-thirds of US-produced films youth rated and government subsidized in Britain, Canada, or the US, contain smoking. Despite the fact that the economic benefits of subsidies have been questioned [37], the political power of the multinational media companies that lobby for them makes it likely that governments will continue to provide them, our analysis suggest that they can seriously undermine tobacco control efforts by indirectly promoting smoking and, hence, tobacco sales. The FCTC defines “tobacco advertising and promotion” as “any form of commercial communication, recommendation or action with the aim, effect or likely effect of promoting a tobacco product or tobacco use either directly or indirectly.” [8]. Because these subsidies indirectly promote tobacco use through media, they represent a violation of FCTC Article 13, which requires countries to “undertake a comprehensive ban of all tobacco advertising, promotion and sponsorship” [8]. (The UK and Canada, but not the US, have ratified the FCTC.) Governments should ensure that film subsidy programmes are harmonised with public health goals by making films with tobacco imagery ineligible for public subsidies. Such action will incur no costs to governments and should be an attractive policy option, particularly in the current financial climate.

**Table 4:** Louisiana (US) tax credits for wide-release films, by rating and tobacco imagery, 2006–2008.

Year	Louisiana Tax Credits (US$ millions)					Percent of Tax Credits for Youth-Rated Films with Smoking
	Youth Rated		R Rated		Total	
	Smoking	Smoke-Free	Smoking	Smoke-Free		
2006	13	13	4	—	30	43
2007	31	18	8	—	57	54
2008	18	13	11	0.15	43	42
**Total**	62	44	23	0.15	129	48

Tax credits estimated at 25% of in-state spend, per film, by Louisiana Economic Development. Totals may not equal components because of rounding.

## Abbreviations

BBFC: British Board of Film Classification
FCTC: Framework Convention on Tobacco Control
MPAA: Motion Picture Association of America
WHO: World Health Organization
